# Appropriateness of referrals from primary care for lumbar MRI

**DOI:** 10.1186/s12998-022-00418-4

**Published:** 2022-02-22

**Authors:** Susanne Brogaard Krogh, Tue Secher Jensen, Nanna Rolving, Janus Nikolaj Laust Thomsen, Casper Brink Hansen, Christoffer Høj Werenberg, Erik Rasmussen, Rune Carlson, Rikke Krüger Jensen

**Affiliations:** 1 Department of Diagnostic Imaging, Silkeborg Regional Hospital, Silkeborg, Denmark; 2 Department of Sports Science and Clinical Biomechanics, University of Southern Denmark, Odense, Denmark; 3 Chiropractic Knowledge Hub, Odense, Denmark; 4 DEFACTUM, Central Denmark Region, Aarhus, Denmark; 5 Center for General Practice, Department of Clinical Medicine, Aalborg University, Aalborg, Denmark

**Keywords:** Low back pain, MRI, Imaging appropriateness

## Abstract

**Background:**

International guidelines do not recommend routine imaging, including magnetic resonance imaging (MRI), and seek to guide clinicians only to refer for imaging based on specific indications. Despite this, several studies show an increase in the use of MRI among patients with low back pain (LBP) and an imbalance between appropriate versus inappropriate use of MRI for LBP. This study aimed to investigate to what extent referrals from general practice for lumbar MRI complied with clinical guideline recommendations in a Danish setting, contributing to the understanding and approaches to lumbar MRI for all clinicians managing LBP in the primary sector.

**Materials and methods:**

From 2014 to 2018, all referrals for lumbar MRI were included from general practitioners in the Central Denmark Region for diagnostic imaging at a public regional hospital. A modified version of the American College of Radiology Imaging Appropriateness Criteria for LBP was used to classify referrals as appropriate or inappropriate, based on the unstructured text in the GPs’ referrals. Appropriate referrals included fractures, cancer, symptoms persisting for more than 6 weeks of non-surgical treatment, previous surgery, candidate for surgery or suspicion of cauda equina. Inappropriate referrals were sub-classified as lacking information about previous non-surgical treatment and duration.

**Results:**

Of the 3772 retrieved referrals for MRI of the lumbar spine, 55% were selected and a total of 2051 referrals were categorised. Approximately one quarter (24.5%) were categorised as appropriate, and 75.5% were deemed inappropriate. 51% of the inappropriate referrals lacked information about previous non-surgical treatment, and 49% had no information about the duration of non-surgical treatment. Apart from minor yearly fluctuations, there was no change in the distribution of appropriate and inappropriate MRI referrals from 2014 to 2018.

**Conclusion:**

The majority of lumbar MRI referrals (75.5%) from general practitioners for lumbar MRI did not fulfil the ACR Imaging Appropriateness Criteria for LBP based on the unstructured text of their referrals. There is a need for referrers to include all guideline-relevant information in referrals for imaging. More research is needed to determine whether this is due to patients not fulfilling guideline recommendations or simply the content of the referrals.

## Background

Low back pain (LBP) is one of the most common reasons for patients to seek care in primary care both nationally and internationally [1, 2]. Management strategies for LBP are based on an evaluation of the individual patient, including case history, physical examination and in some cases imaging. To ensure the best patient care and to optimise management of LBP, several clinical recommendations have been developed, such as those from the American College of Radiology (ACR) Appropriateness Criteria for Low Back Pain [3], the NICE imaging guidelines [4] and the Danish national imaging guidelines [5]. These recommendations are all evidence-based to promote the best clinical practice, including when it is appropriate to use imaging. Most patients with acute LBP, with or without radiculopathy, have substantial improvements in function and pain within the first 4 weeks, and the guidelines do not recommend routine imaging for those patients [3–5]. In general, imaging is considered for patients with approximately 6 weeks of non-surgical treatment where there has been little or no improvement in their LBP [6]. Furthermore, guidelines recommend that imaging is always considered for patients with suspicion of serious underlying conditions, such as cauda equina syndrome, malignancy, fracture, and infection, i.e. red flag signs and symptoms [6].

Imaging is an important driver of the health care costs associated with LBP, not only because of the direct costs of the test procedures but also because of the downstream effects. Unnecessary imaging can lead to additional tests, consultations, referrals and may even result in invasive procedures of limited or questionable benefit [7]. In addition, unnecessary imaging can be harmful as incidental findings can lead to worry or concern in patients [8] and increase the risk of adverse outcomes (e.g., absence from work) [9]. Early MRI in patients with non-specific LBP has been shown to result in more back surgery, increased use of opioids and a higher pain score [10]. Despite a clear recommendation against routine imaging of non-specific LBP, this is not well implemented in clinical practice. A systematic review from 2018 showed that 35% of patients referred for lumbar imaging were judged inappropriate due to the absence of red flags for serious pathology, and 32% were judged inappropriate based on the criteria of no clinical suspicion of pathology [11]. Furthermore, a systematic review from 2019 reported that 25% of patients with LBP in primary care underwent imaging and that the use of complex imaging (including MRI) for LBP had increased over the previous 2 decades [12]. In the Danish national setting, the same pattern of increasing use of MRI was reported, with a 20% increase from 2013 to 2018 (from 70,310 to 2013 to 84,124 in 2018) in patients aged 15–85+ years [13]. However, it is unclear to what extent MRI referrals in Denmark are adhering to guideline recommendations. Therefore, this study aimed to investigate how MRI referrals for LBP from general practice clinics in a Danish primary care setting complied with current guideline recommendations, contributing to the understanding and approaches to lumbar MRI for all clinicians managing LBP in the primary sector.

### Objective

To investigate the proportions of appropriate and inappropriate referrals, according to ACR guidelines, for lumbar spine MRI from general practice over a period of 5 years.

## Method

This study is reported in accordance with the ‘Strengthening the Reporting of Observational Studies in Epidemiology’ (STROBE) statement [14].

### Design

This was a retrospective cross-sectional study.

### Setting

In Denmark, general practitioners (GPs) and chiropractors can refer patients with spinal pain for an MRI at a radiology department at a publicly funded hospital. However, while visits to GPs are free of charge, seeing a chiropractor is only reimbursed by approximately 20% (in average). As referrals from chiropractors merely accounted for 8% of the total referrals available and to ensure homogeneity in the dataset, referrals from chiropractors along with referrals from the secondary sector was excluded. When referred for an MRI, citizens are free to choose any public hospital, but most patients will choose the local hospital. Data for this study were collected at the Department of Diagnostic Imaging, Silkeborg Regional Hospital in Central Denmark Region from January 1st, 2014 to December 31st, 2018. The Department primarily receives referrals from the municipality of Silkeborg which is the 10th largest of the 98 municipalities in Denmark, with a population of 94,026 (1st of January 2020).

### Study population

The study population consisted of referrals from general practice clinics received by the radiology department for patients with LBP referred for a lumbar spinal MRI in the period 2014–2018. These referrals were identified by a unique identification number used in the electronic transmission process. This ensured that the sample did not include other primary care providers or hospital departments, such as chiropractors, who have had the right to refer patients for MRI in Denmark since January1st 2014. The referrals for an MRI of the lumbar spine concerned patients ≥ 18 years of age with LBP, with or without radiating leg pain. The referrals were received by the radiology department and checked for contraindications for MRI. During the data collection period, there were no other criteria for accepting or declining the MRI referral, and according to personal communication with the department, almost all referrals were accepted even if they did not comply with general national guidelines for referral. Referrals were only declined if they did not contain enough information about absolute MRI contraindications, such as non-MRI compliant materials, implants or devices.

### Variables

**Fig. 1:**
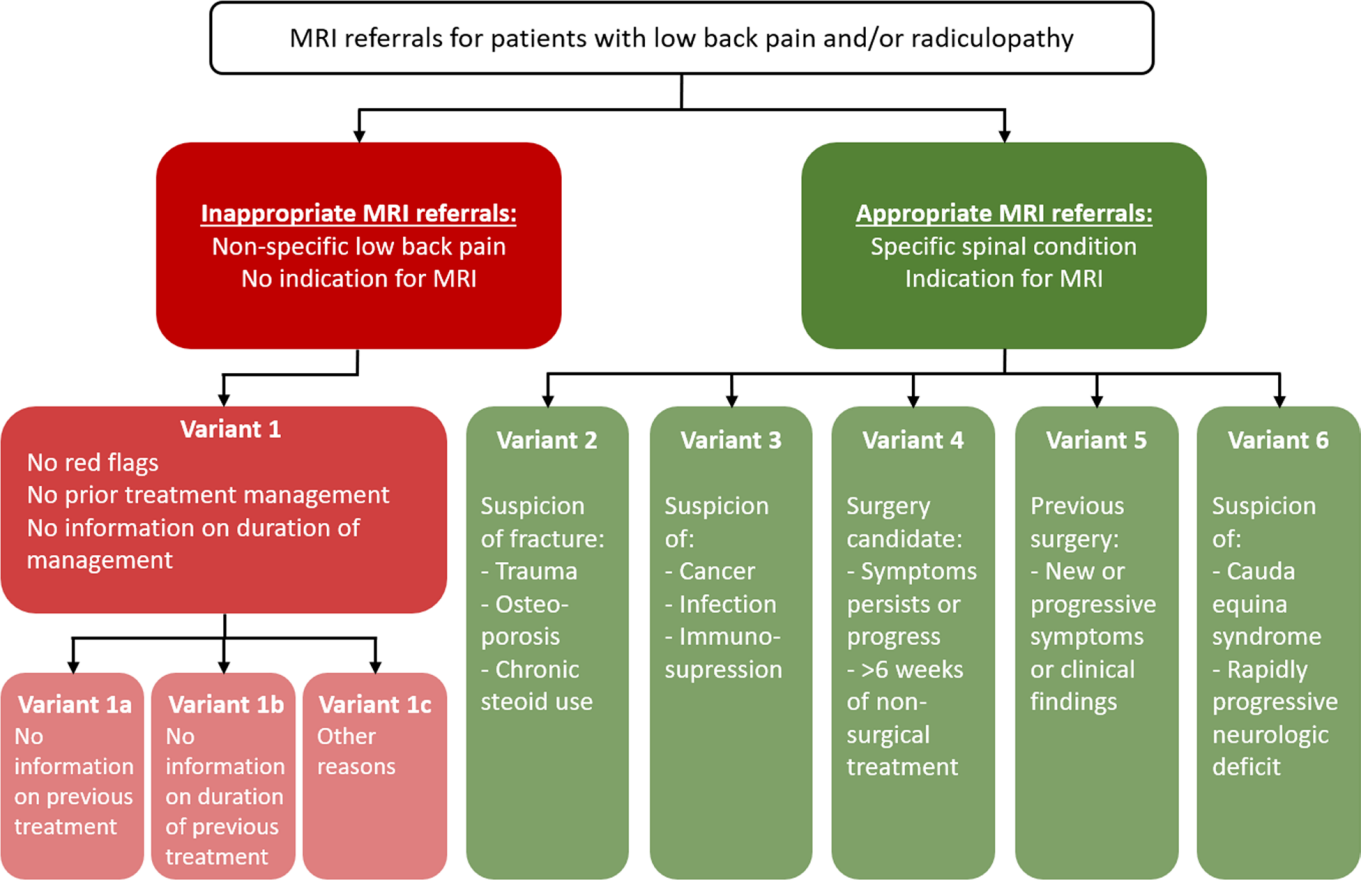
Flow chart for the classification of the referrals according to the modified ACR imaging appropriateness criteria

An MRI imaging referral is required to contain information about MRI contraindications and a narrative text, including a short summary of the patient’s medical history explaining why the referral is relevant according to current guidelines. Based on the narrative text, referrals were classified as compliant (appropriate) or non-compliant (in-appropriate) according to the 2015 version of the ACR Imaging Appropriateness Criteria for LBP [3]. These criteria (Fig. 1) describe five variants of appropriate MRI referrals (‘Variants 2–6’): (Variant 2) Suspicion of fracture (e.g. trauma, osteoporosis, chronic steroid use); (Variant 3) Suspicion of cancer, infection, immunosuppression or spondyloarthritis; (Variant 4) Candidate for surgery or intervention with persistent or progressive symptoms during or following 6 weeks of conservative management; (Variant 5) New or progressing symptoms or clinical findings with a history of prior lumbar surgery; Variant 6) Suspected cauda equina syndrome or rapidly progressive neurological deficit. If the MRI referrals did not include information on any of the above conditions, the referrals were deemed inappropriate (Variant 1). The inappropriate referrals were divided into three subcategories: (1a) no information on previous non-surgical treatment, (1b) no information on the duration of non-surgical treatment, or (1c) other reasons (e.g., errors such as missing narrative text, referrals of cervical/thoracic spine).

In addition to the classification of appropriateness, information on demographic data, including the patient’s age and sex, zip code of their residential address, zip code of the referring GP’s clinic, MRI referral date and MRI procedure including body region, were collected.

### Data collection

MRI referrals were received and stored in the Kodak Carestream RIS (Radiology Information System) version 6.3.0 (Carestream Health, NY, USA). The narrative texts were exported from the RIS archive and were de-identified and uploaded to REDCap electronic data capture tools hosted at Aarhus University REDCap (Research Electronic Data Capture) [15, 16]. Demographic data were extracted from the RIS data. The narrative referral text was used to assess its appropriateness. Five raters participated in the data collection (SBK, CBH, CHW, ER and RC). Each rater entered the classification of the referrals into an Excel spread sheet (Microsoft Excel 2010, Microsoft Corporation, Redmond, WA, USA), developed for the purpose of this study based on Fig. 1 and designed to allow only one category per referral to be entered. Details of this process have been described elsewhere [17] The full dataset was randomly divided into five datasets of equal size by administrative staff in the radiology department who were not part of the study. Due to logistical and time constraints, only a sample representing approximately half of the referrals could be assessed. Prior to data collection, the inter-rater reliability was tested between the five raters. Inter-tester reliability was ‘Substantial’ to ‘Almost perfect’ with Kappa values ranging from 0.76 (95% CI 0.55–0.89) to 0.82 (95% CI 0.72–0.92) depending on the number of subcategories [14].

### Data management and analysis

The appropriate and inappropriate referrals were reported in percentages for the overall categories and for the subcategories. The development over the 5-year period was shown graphically by calculating the proportions per year of the two main categories.

Additional variables were presented in tabular form with summarising tables. Baseline characteristics of referred patients were compared between the randomly selected and classified referrals and those not classified. Data management and statistical analysis were performed using Stata version 16 (StataCorp, College Station, Texas, USA).

## Results

**Fig. 2:**
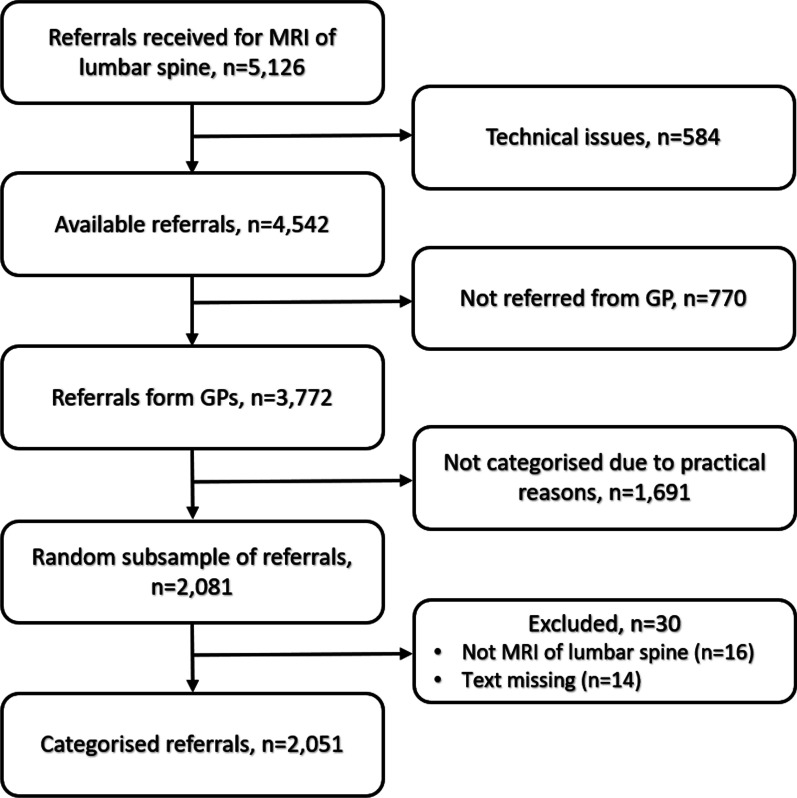
Flow of the selection and classification process

A total of 4542 referrals for MRI of the lumbar spine were retrieved. Referrals from general practice clinics accounted for 3772 (83%) and of these, 2081 (55%) were allocated for classification. Finally, a total of 2051 referrals were categorised (Fig. 2).

We excluded 584 referrals for technical reasons, e.g. because of incomplete referral forms, or that the referral text was sent in a separate document and therefore not accessible without compromising patient anonymity. To ensure data homogeneity, we only included referrals from GPs leaving 3772 potential referrals to be categorised. Due to logistical reasons and time constraints, only a sample representing approximately half of the referrals were assessed (n = 2081). The sample of referrals were randomly selected, and we made no attempt to include an equal proportion per year or stratify by demographics. However, as mentioned in the results, we found no differences between the proportions of categorised and not-categorised referrals with respect to age, sex, year of referral or municipality, please see Table 1.

**Table 1:** Baseline characteristics of referred patients by classification status

	Classified	Not classified	Total	*P* value	Missing/N (Pct)
n (%)	2051 (54.8)	1691 (45.2)	3742 (100)		0/3742 (0.00)
Age, mean (SD)	54.1 (15.8)	54.8 (15.5)	54.4 (15.6)	0.20	0/3742 (0.00)
Sex, n (%)					
Men	925 (45.1)	749 (44.3)	1674 (44.7)		
Women	1126 (54.9)	942 (55.7)	2068 (55.3)	0.62	0/3742 (0.00)
Year, n (%)					
2014	266 (13.0)	218 (12.9)	484 (12.9)		
2015	220 (10.7)	172 (10.2)	392 (10.5)		
2016	344 (16.8)	271 (16.0)	615 (16.4)		
2017	608 (29.6)	498 (29.5)	1106 (29.6)		
2018	613 (29.9)	532 (31.5)	1145 (30.6)	0.85	0/3742 (0.00)
Zip code, n (%)					
Outside Eastern Jutland	155 (7.7)	107 (6.4)	262 (7.1)		
Eastern Jutland	1850 (92.3)	1554 (93.6)	3404 (92.9)	0.13	76/3742 (2.03)

In the data collection period from 2014 to 2018, a total of 104 general practice clinics referred the 2051 patients to MRI. The majority of referrals were from general practice clinics in the municipality of ‘Silkeborg’ (68%) and the rest were from other municipalities in the Central Denmark Region, except for eight referrals (0.39%) received from outside the Region.

**Fig. 3:**
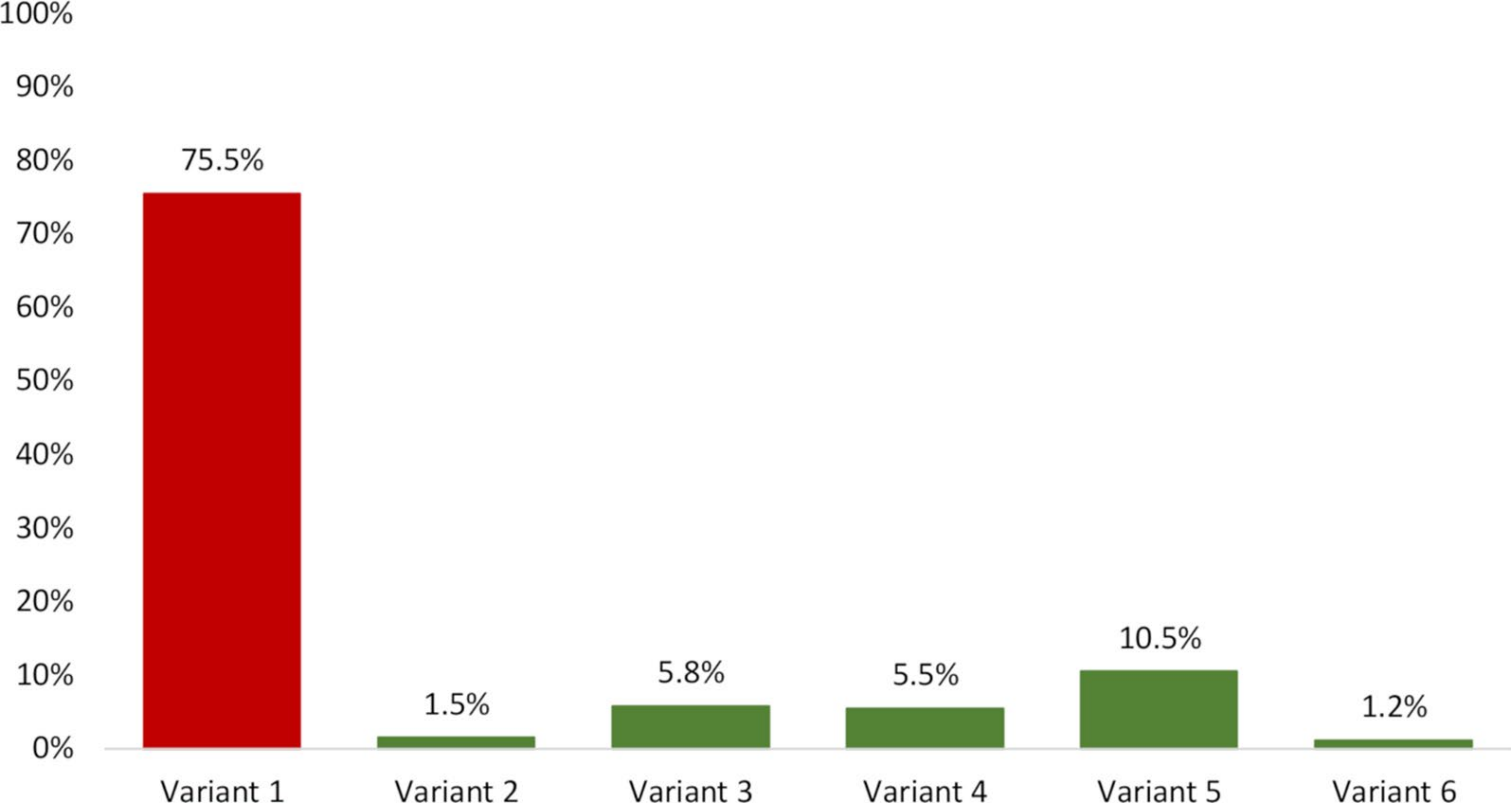
Classification of the referrals according to the modified ACR imaging appropriateness criteria

Overall, 1548 (75.5%) of the referrals from general practice clinics did not fulfil the ACR criteria for appropriate referrals, whereas 503 (24.5%) were categorised into one of the five appropriate variants (see Fig. 1). Of the five appropriate variants, Variant 5 ‘New or progressing symptoms or clinical findings with history of prior lumbar surgery’ was the most common category (10.5%) followed by Variant 3 ‘Suspicion of cancer, infection, immunosuppression or spondyloarthritis’ (5.8%) and Variant 4 ‘Candidate for surgery or intervention with persistent or progressive symptoms during or following 6 weeks of conservative management’ (5.5%) (Fig. 3). Of the 1548 inappropriate referrals, 782 (50.5%) were subcategorised as Variant 1a because there was no information on previous non-surgical treatment, and 763 (49.3%) as Variant 1b because there was no information on the duration of non-surgical treatment. In three referrals subclassified as 1c’ Other reasons,‘ the text explained that the patient could not afford or did not wish to engage in conservative care.

Inappropriate referrals: (Variant 1) Inappropriate referral. Appropriate referrals: (Variant 2) Suspicion of fracture; (Variant 3) Suspicion of cancer, infection, immunosuppression or spondyloarthritis; (Variant 4) Candidate for surgery or intervention with persistent or progressive symptoms during or following 6 weeks of conservative management; (Variant 5) New or progressing symptoms or clinical findings with a history of prior lumbar surgery; (Variant 6) Suspected cauda equina syndrome or rapidly progressive neurological deficit. The categories of appropriate referrals were mostly related to the patient being a candidate for surgery or worsening of the patients’ known symptoms (Variant 4 and Variant 5), while suspicion of cancer or spondyloarthritis (Variant 3) where the most common pathological reasons for referral.

For referrals categorised as inappropriate, the mean age of patients was 53.3 years (SD 15.65) (range 18–94), and 54.7% were women, and for appropriate referrals, the mean age was 56.4 years (SD 16.0) (range 19–90), and 55.5% were women. The sex of the patient was almost evenly distributed within the appropriate referral categories except for the two smallest categories: Variant 2 ‘Suspicion of fracture (e.g., trauma, osteoporosis, chronic steroid use)’ with 71.0% women (n = 22) and Variant 6 ‘Suspected cauda equina syndrome or rapidly progressive neurological deficit’ with 66% women (n = 16). Variant 2 had the oldest patients with a mean age of 67.7 years (SD 11.2) and Variant 4 ‘Candidate for surgery or intervention with persistent or progressive symptoms during or following 6 weeks of conservative management’ the youngest with a mean age of 49.3 years (SD 16.3). (Table 2).

**Table 2:** Characteristics of referred patients by categorisation status (both appropriate and inappropriate referral variants)

	n	Mean age (SD)	Age range	Women (%)	Men (%)
Variant 1	1548	53.4 (15.7)	18–94	54.72	45.28
Variant 2	31	67.7 (11.2)	43–85	70.97	29.03
Variant 3	119	55.9 (17.2)	19–90	52.10	47.90
Variant 4	113	49.4 (16.3)	21–85	51.33	48.67
Variant 5	216	59.0 (14.1)	19–87	56.02	43.98
Variant 6	24	53.3 (16.4)	19–81	66.67	33.33

Inappropriate: (Variant 1) Inappropriate referral; Appropriate: (Variant 2) Suspicion of fracture; (Variant 3) Suspicion of cancer, infection, immunosuppression or spondyloarthritis; (Variant 4) Candidate for surgery or intervention with persistent or progressive symptoms during or following 6 weeks of conservative management; (Variant 5) New or progressing symptoms or clinical findings with a history of prior lumbar surgery; (Variant 6) Suspected cauda equina syndrome or rapidly progressive neurological deficit.

**Fig. 4:**
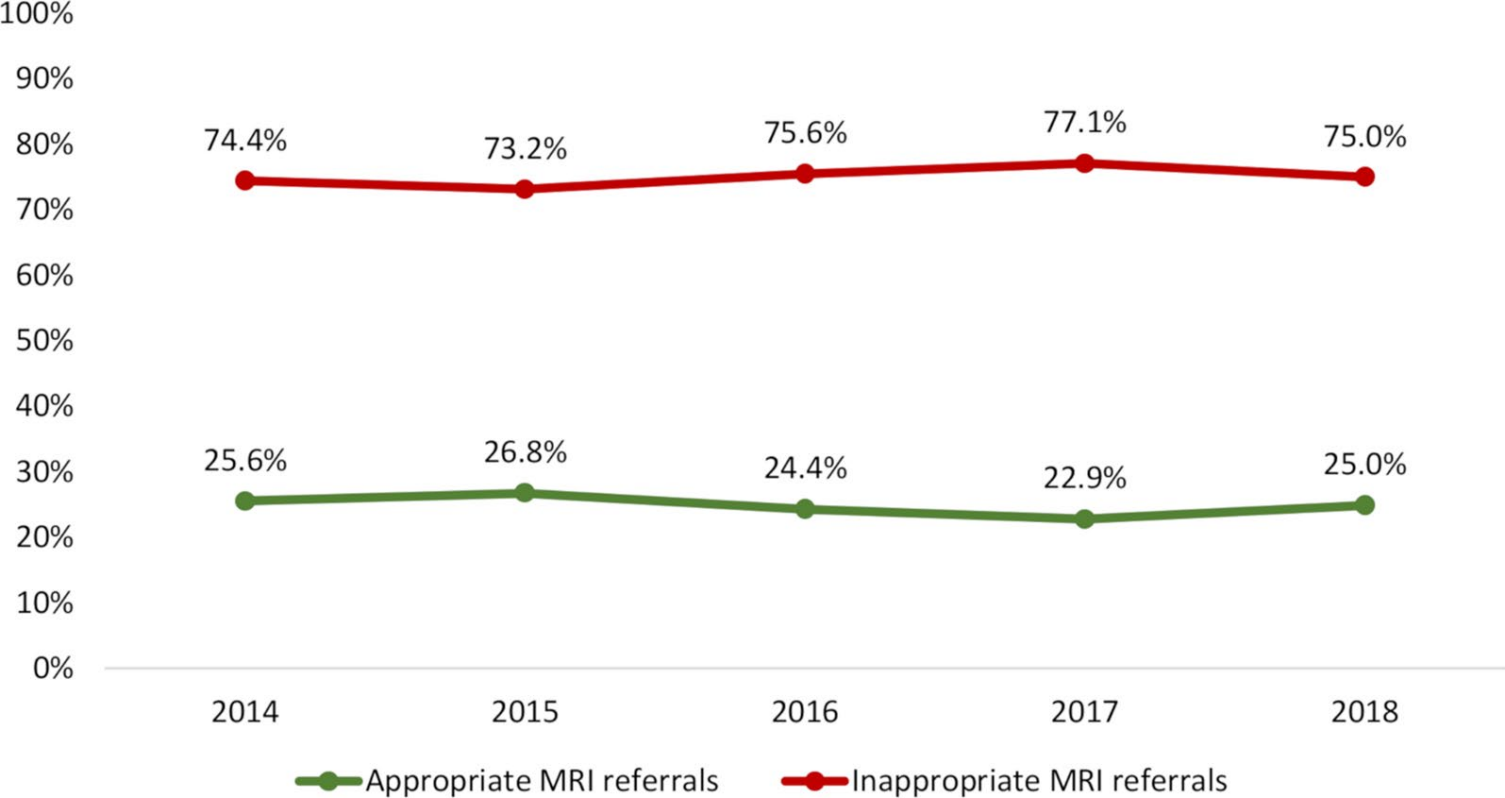
Trajectory of appropriate and inappropriate MRI referrals from 2014 to 2018

Apart from a statistically insignificant yearly fluctuation, the distribution of appropriate and inappropriate MRI referrals remained stable from 2014 to 2018 (Fig. 4). There were no differences in mean age or sex distribution per year.

## Discussion

### Main findings

This study classified 2051 lumbar MRI referrals as compliant or non-compliant with the 2015 version of the ACR Imaging Appropriateness Criteria for LBP [3]. The classification was based on the narrative text from the MRI referral, and no patients were clinically evaluated in this study.

Three-quarters (75.5%) of the MRI referrals were deemed inappropriate, and 24.5% were classified as appropriate. The distribution was stable from 2014 to 2018. To our knowledge, no active implementation strategies targeting imaging for spinal pain conditions in general practice were carried out during that period.

### Comparison with other studies

Many studies have investigated the appropriateness of lumbar MRI, and a variety of methods have been used [11, 18–21]. Some studies used imaging guidelines to clarify the appropriateness of MRI, and others used clinical symptoms and/or various standards of red flags [11, 22] The considerable variation in methods for classification of appropriateness of MRI makes it difficult to make comparisons between studies. An Australian systematic review from 2018 estimates the overuse and underuse of imaging in the management of LBP [11]. The review included 33 studies and assessed the use of X-ray, CT and/or MRI imaging referrals for patients presenting for care. Inappropriate referrals in people referred for imaging were assessed in 23 studies and showed a pooled effect of 34.8% when ‘Absence of red flag clinical features’ was used as the inappropriateness criterion and 31.6% when ‘No clinical suspicion of pathology’ was the criterion. The majority of studies assessed patients referred from general practitice. Four studies [11, 23–25] assessed imaging referrals received by radiology departments from primary care physicians, which we considered were comparable to the setting of our study. Rates of inappropriate referrals ranged from 20 to 47.9% which were considerably lower than the 75.5% found in our study. One study [26] used the same ACR imaging guideline as in our study to assess appropriateness of MRI referrals and reported the highest rate of inappropriate referrals (47.9%) of the four studies.

A scoping review from 2019 included 23 studies describing adult LBP imaging appropriateness in general practice [22]. A range of red flag features was utilised to determine imaging appropriateness. Most studies considered appropriateness in a binary manner by the presence of any red flag feature. Ten different guidelines were referenced in 16 of the 23 studies, while seven studies (30%) used combined methods or modified guidelines. The method for calculating the proportion of inappropriate imaging varied. 10% of the studies used the total number of patients presenting with LBP as the denominator, suggesting most studies underestimated the rate of inappropriate imaging and did not capture where imaging was not performed for clinically suspicious LBP.

Both the Australian systematic review from 2018 [11] and the scoping review from 2019 [22] conclude that many different methodologies are used to assess LBP imaging appropriateness. 10% of the studies used clinical data to calculate if an MRI was appropriate, which is a different way of calculating appropriateness compared to the current study that uses referrals to calculate appropriateness. Therefore, results cannot be directly compared as the proportion of appropriate MRIs uses different denominators. The current study is therefore not directly comparable to the studies mentioned above. Although they are based on data from MRI referrals, none of them uses the same guideline or checklist to classify referrals, and they do not report the same considerably high number (75.5%) of inappropriate MRI referrals as was found in our study. This large proportion of inappropriate referrals in the current study was probably due to the strict criteria for appropriateness. Only precise information on previous non-surgical treatment and the duration of that treatment in the referral text was used in the evaluation of appropriateness. If information about previous treatment or duration was not provided, the referral was classified as inappropriate, even if text like “*the patients have for some time maintained training*” was available. In cases like this, it was unclear what type or level of training had been performed or what period of time was involved (more or less than 6 weeks), which is essential information in the evaluation of appropriateness when using the ACR imaging guideline. If the criterion of duration was omitted, 38.3% (75.5 minus 37.2%) of the MRI referrals would have been inappropriate which is still higher but closer to the results reported in the previously mentioned literature [11, 22]. It is possible that important clinical symptoms were absent due to oversight in the referrals or that information about non-surgical treatment or duration was not mentioned because of a lack of knowledge about imaging referral guidelines for LBP. Furthermore, there was a tradition in the department of accepting all referrals despite the lack of information and no feedback procedures existed to inform the GPs that some referrals lacked proper information. This could partly explain why important information was not included in the referrals, which led to a substantially higher number (75.5%) of inappropriate MRI referrals in our study.

### Demographic data

More than half of the appropriate referrals were categorised as either (Variant 4) ‘Candidate for surgery or intervention with persistent or progressive symptoms during or following 6 weeks of conservative management’ or (Variant 5) ‘New or progressing symptoms or clinical findings with a history of prior lumbar surgery’. Both variants are related to clinical management rather than suspicion of serious pathology. In the current study, only 8.5% of all referrals (35% of the appropriate referrals) were referred due to suspicion of serious pathology as in Variants 2, 3 or 6 (fracture, infection, cancer, or cauda equina). In comparison, a study by Gidwani et al. [20] found that 24% of the appropriate MRIs for LBP (n = 76.663) had suspicion of red flag conditions as identified by diagnostic codes (IDC-9-CM). Mean age and sex distribution were relatively comparable between the six variants (Table 2). Only Variant 2 (Suspicion of fracture) had a higher percentage of women (70.97%) compared with men (29.03%), and mean age (67.7) was higher when compared to the other variants. This finding is in line with an increasing risk of osteoporotic fractures among women with increasing age.

### Perspectives

This study focused on referrals for MRIs of the lumbar spine, in which the narrative text of the referrals was not compared with clinical data. Therefore, it is not possible to determine if a referred patient truly had indications for MRI. Also, it is not possible to measure the proportion of cases where clinical symptoms indicated appropriate MRI but where MRI was not performed (underuse). However, the study reflects what referrals look like in a Danish clinical setting, and it shows that these were not aligned with clinical guideline recommendations. To investigate the ‘true’ prevalence of guideline-appropriate referrals, future research should contain clinical information at the patient level, including precise information of duration and type of non-surgical treatment. Ideally, appropriate referrals should demonstrate high sensitivity for the detection of serious pathology with a reasonably high specificity to limit unnecessary imaging of patients without serious pathology.

Danish National Clinical Guidelines for non-surgical treatment of patients with recent onset LBP or lumbar radiculopathy [27] was published in 2016. Although it recommends against routine use of MRI for LBP with or without radiculopathy, there was no change in the distribution of appropriate and inappropriate MRI referrals from 2014 to 2018. From a clinical perspective, it seems timely to develop an implementation strategy of imaging guidelines for LBP among GPs to ensure that only patients with a clear indication for MRI are referred and to increase the quality of the referrals.

### Methodological considerations

From a clinical perspective, it seems timely to develop an implementation strategy for spinal imaging among GPs to ensure guidelines compliant MRI referrals which potentially could provide the right diagnostic imaging modality to the right patient at the right time. To ensure that clinicians who refer patients to imaging include all guidelines relevant information in the referral forms would require a thorough dissemination and implementation of clinical practice guidelines, and an evaluation of the implications to clinical practice.

The strengths of this study are the large study sample and the use of a previously tested data collection method that had shown substantial to high inter-rater reliability. The data collection method was based on a well-documented international method for the assessment of imaging guideline appropriateness (ACR) [3]. Although the ACR is developed in the US and that there are differences in both populations and health care systems of the US and Denmark, we consider that these differences are likely to be minor with respect to the present study, as the overall recommendations for the use of diagnostic imaging for low back pain conditions in Denmark [27] are very similar to the ACR criteria.

To ensure a homogeneous practice of referral from general practice clinics and triage by the receiving department, the data were collected from a single imaging department located at a medium-sized hospital with a catchment that included both city and rural areas. However, this could at the same time be a weakness as it is possible that local routines and agreements exist that are not applicable to other parts of the country.

In everyday clinical practice, imaging referrals are not controlled and monitored against clinical findings and symptoms. Although this study cannot tell if the patients had an appropriate indication that was not reflected in the narrative text of the referral, the results do reflect that in everyday practice there is no evidence that guidelines are implemented in either GP referrals or at the receiving imaging department.

## Conclusions

The majority of lumbar MRI referrals (75.5%) from general practitioners for lumbar MRI did not fulfil the ACR Imaging Appropriateness Criteria for LBP according to the unstructured text of the referrals. This study cannot determine whether this is due to patients not fulfilling the criteria or to the content of the information in the referrals. There is a need for those referring patients to include all guideline-relevant information in the referrals for imaging. More research is needed to determine whether this is due to patients not fulfilling guideline recommendations or simply to the content of the referrals.

## Data Availability

The dataset used in the current study is available from the corresponding author on reasonable request.
